# Adsorption and desorption of nutrients from abattoir wastewater: modelling and comparison of rice, coconut and coffee husk biochar

**DOI:** 10.1016/j.heliyon.2021.e08458

**Published:** 2021-11-23

**Authors:** Morris Konneh, Simon M. Wandera, Sylvia I. Murunga, James M. Raude

**Affiliations:** a Pan African University for Basic Sciences, Technology and Innovation (PAUSTI), P.O. Box 62000-00200, Nairobi, Kenya; b Department of Civil, Construction and Environmental Engineering, Jomo Kenyatta University of Agriculture and Technology, P.O. Box 62000-00200, Nairobi, Kenya; c Agricultural and Biosystems Engineering Department (ABED), Jomo Kenyatta University of Agriculture and Technology, P.O. Box 62000-00200, Nairobi, Kenya; d Soil, Water and Environmental Engineering Department (SWEED), Jomo Kenyatta University of Agriculture and Technology, P.O. Box 62000-00200, Nairobi, Kenya

**Keywords:** Biochar, Adsorption, Desorption, Langmuir, Freundlich, Nitrates, Nitrites, Slaughterhouse

## Abstract

Enrichment of water bodies with nutrients from wastewater is one of the causes of eutrophication to aquatic ecosystems. This study investigated the use of biochar derived from rice husk, coconut husk, and coffee husk in adsorbing nitrates (NO3-N) and nitrites (NO2-N) from slaughterhouse wastewater. It also explored the desorption efficiencies of the adsorbed nutrients to ascertain the applicability of the enriched biochars as slow-release fertilizers. To characterize the physicochemical properties of the biochars, scanning electron microscopy (SEM) was used. Fourier transforms infrared spectroscopy (FTIR), elemental analysis (CHNO) Langmuir and Freundlich, and the isotherm models were employed to fit the experimental equilibrium adsorption data. It was observed that the Langmuir isotherm model has the best fit of NO3- N and NO2- N on all the biochars. And this was based on the coefficient of correlation values. Also, the coconut husk biochar has the highest adsorption capacities of NO3-N and NO2-N at 12.97 mg/g, and 0.244 mg/g, respectively, attributing to its high porosity as revealed by the SEM images. The adsorption capacities for the rice husk char were 12.315 and 0.233 mg/g, while that for coffee husk char were12.08 mg/g and 0.218 mg/g for NO3-N and NO2-N, respectively. The relatively higher amount of NO3-N adsorbed to that of NO2-N could be attributed to its higher initial concentration in the solution than nitrite concentration. The desorption efficiencies of nitrates were 22.4, 24.39, and 16.79 %, for rice husk char, coconut husk char and coffee husk char, respectively. For the rice husk char, coconut husk char and coffee husk char, the nitrites desorption efficiencies were 80.73, 91.39, and 83.62 %, respectively. These values are good indicators that the studied biochar can be enriched with NO3- N and NO2- N and used as slow-release fertilizers.

## 1 Introduction

The most prominent factors that have resulted in an overabundance of municipal, industrial, and agricultural waste in human civilization are rapid population increase, industrial development, urbanized culture dissemination, and excessive material consumption. In recent years, improper garbage disposal has resulted in several environmental threats and crises in human society. Slaughterhouses are a major source of water pollution globally. Although environmental authorities frequently monitor point sources of pollution, such as effluents from municipal wastewater treatment facilities and industry, abattoirs have significant challenges in terms of monitoring and enforcement, posing a threat to aquatic life and natural water quality (Quarters et al., 2018).

Nitrogen compounds constitute one of the major nutrients in wastewater effluents from slaughterhouses (Michael et al., 2020). According to Mossa Hosseini et al. (2011), Nitrates (NO_3_^-^N) not only accelerate excessive plant growth in water bodies but are easily reduced to nitrites (NO_2_^-^N), which poses several health threats to humans such as liver damage and even cancer. Due to their negative environmental effects, efficient technologies for their elimination have attracted much attention.

Among the techniques that have been applied for nitrates removal from water and wastewater are reverse osmosis, electro-dialysis, ion exchange, biological denitrification, chemical denitrification and adsorption (Mohsenipour et al., 2014). However, some weaknesses have been reported among these methods. For instance, ion exchange, reverse osmosis and electro-dialysis, require frequent regeneration of the medium and further treatment for the secondary waste produced (Soares et al., 2008). Biological approaches, on the other hand, have a high operational sensitivity and are influenced by the leachate's physicochemical and biological variability. The toxicity of leachate particularly that of ammonia, may pose a threat to the microbial fauna necessary for anaerobic decomposition, necessitating heating or cooling Ineffectiveness (Lippi et al., 2018). Adsorption has been suggested as an effective method for removing contaminants from polluted media (Badruddoza et al., 2013; Nageeb, 2013). The adsorption process may be more encouraging when agricultural wastes are utilized as adsorbents because they are renewable, low cost, and highly available (Omo-Okoro et al., 2018). Adsorption materials that have been explored for nitrates and nitrites removal from aqueous solutions can be grouped into biochars from agricultural wastes, geological materials (zeolites, clays), slag and fly ash (Bhatnagar and Sillanpää, 2011). The use of biochar for nutrient adsorption has recently attracted wider attention (Ajmal et al., 2020; Otieno et al., 2021; Thao et al., 2021). Production of biochar from agricultural wastes has been preferred due to their abundance, free cost and non-toxicity besides improving waste management (Tan et al., 2015). Biochar is a carbon-rich material produced as a result of pyrolysis of biomass material under limited or no oxygen conditions (Mulabagal et al., 2020; Tan et al., 2015). The resultant char from pyrolysis is porous as a result of loss of volatiles thus making the material to be efficient in adsorption. The enriched biochar after adsorption of nutrients can be used as a slow-release fertilizer to increase soil fertility and sequester carbon (Yao et al., 2011).

This study focused on evaluating the potential of using biochar from rice husks, coconut husks and coffee husks in adsorbing nutrients from slaughterhouse wastewater and subsequent desorption to utilize it in agricultural production.

## 2 Materials and methods

### 2.1 Materials

Slaughterhouse wastewater was collected in sterilized glass sample bottles from a slaughterhouse in Juja, Kiambu County, Kenya. The sample was transported in a cooler box and stored in a refrigerator at 6 °C to minimize deterioration of the physicochemical properties before use in experiments. Before experimentation, the wastewater was centrifuged and then filtered using Whatman filter paper No. 42 to eliminate hindrance from suspended solids. The biochars used in this study were from three agricultural biomass materials (viz., rice husk, coconut husk, and coffee husk), considering their easy availability and low cost. They were produced under slow pyrolysis at 700 °C and residence time of 2 hours in a reactor. To ensure an oxygen-limited environment during pyrolysis, nitrogen gas was supplied to the reactor. At the end of pyrolysis, the chars were allowed to cool in the reactor to temperatures below 100 °C and subsequently transferred into a desiccator for final cooling. The chars were washed with distilled water (8000 mL per batch) to remove ashes and other impurities. The washing was done with biochar particles suspended on the steel mesh (0.5 mm) and distilled water (8000 mL) per batch, flushed from the top.

### 2.2 Materials characterization

Wastewater was characterized for pH using HANNA 211 Microprocessor pH meter, while the conductivity meter was used to determine the electrical conductivity. The COD was determined as per the close reflux method. BOD_5,_ TS, nitrogen compounds (NH_4_^+^N, NO_3_^−^N, NO_2_^−^N) and PO_4_^3−^P were determined as per the American Public Health Association Standard Methods (APHA, 2017) For the metal ions analysis, 100mL of wastewater was filtered using Whatman filter paper No. 1. Aquarregia (1HCl and 3HNO3) was made by combining 1:3 HCl: HNO3 and heated for 15 minutes before cooling to room temperature. 3–5mL of the mixture was added to the sample and heated for 30 minutes to ensure that it was completely digested. The sample was cooled, filtered, and its absorbance was measured. Atomic Absorption Spectrometer (AAS) technique was used to analyze ions of Cd, Ca, Zn, Cr, Ni, Cu, Fe and Pb.

Biochars were characterized for proximate parameters (fixed carbon, volatile matter, moisture content, and ash content) according to ASTM D1762-84 standard analysis for charcoal (McLaughlin, 2018). Elemental characterization (C, H, N, and O) was done using an elemental analyzer (AAS iCE 3300), while pH was measured using a pH-meter (HANNA 211) and the electrical conductivity using a conductivity-meter (Palintest). Scanning Electron Microscopy (AS 08600000-257) was used in the morphological characterization while Fourier transforms infrared spectroscopy (FTIR) was used to study the structural chemical functional groups of the biochar samples within a wavenumber range of 400–4000 cm^−1^.

### 2.3 Batch adsorption experiments

Slaughterhouse wastewater was used in this experiment. Sorption kinetics were evaluated at room temperature 26 °C and the initial pH for the sorption solution was 7.35 ± 0.15. The initial concentration of the Nitrates and Nitrites were measured and recorded. 1.5 g of rice husk biochar, coffee husks biochar and coconut husk biochar were then added into separate containers containing 50 mL each of the slaughterhouse wastewater. The samples were then shaken at 120 rpm in a mechanical shaker. The samples were taken after 30 minutes, 60 minutes, 90 minutes and 120 minutes and analyzed nitrates and nitrites in the residual solution using a Spectrophotometer (Shimadzu UV-1800, Japan). The samples were then centrifuged at 5000 rpm, for 10 minutes and filtered using Whatman filter paper no.42.

The amount of Nitrates and nitrites adsorbed by the biochars were calculated by Eq. (1) as used in previous studies (Kizito et al., 2015; Wang et al., 2015)

(1) %Removed=Co−CtCo×100

Where; C_o_ and C_t_ are the initial concentration of NO_3_^-^N and NO_2_^-^ N and at time t, respectively.

#### 2.3.1 Effects of adsorbent loadings

The experiments were conducted on a batch basis in triplicates. Varying loadings of 0.5g, 1.0g, 1.5g, and 2.0 g in 50 mL of slaughterhouse wastewater at a pH of 7.35 ± 0.15 were used. The samples were shaken at 120 rpm. After shaking for 120 samples were filtered through whatman filter paper no. 42 and the concentrations of nitrates and nitrites determined in the filtrate. The amount of nutrients adsorbed was calculated using Eq. (1).

#### 2.3.2 Effect of pH

Using an adsorbent dosage of 1.5 g in 50 mL solution, the influence of pH on adsorption was investigated by varying the values from 2, 4, 6, 8 and 10 using 0.1M NaOH solution.

### 2.4 Equilibrium isotherm studies

The rate and equilibrium of NO_3_-N and NO_2_-N adsorption in slaughterhouse effluent were investigated using batch equilibrium adsorption studies. Rice, coconut, and coffee husk char adsorbent loadings of 1.5g each were added to 50 mL slaughterhouse wastewater solution, and the pH was adjusted with 0.1M and 0.1NaOH. Using a centrifuge, the utilized adsorbents were separated at the end (5000 rpm, 10 minutes). A spectrophotometer was used to quantify the supernatant after separation (Shimadzu UV-1800, Japan). Finally, using Eq. (2), the nutrients adsorbed (qe, mg/g) and removal efficiency (%) on the chars were calculated.

(2) $$Qe=\frac{C_{o}-C_{e}}{M}\times V$$

Where; C_o_ (mg L^−1^) and C_e_ (mg L^−1^) indicate initial and equilibrium NO_3_^-^N and NO_2_^-^N solution concentrations (mg L^−1^), whereas Qe (mg g^−1^) represents the total ions adsorbed amount per gram (g) of adsorbent at equilibrium, M is the weight of adsorbent (g), and V is the volume of solution (L).

#### 2.4.1 Langmuir isotherm model

This model assumes monolayer adsorption of ions onto homogeneous adsorption sites (Ayawei et al., 2017). It also enables the calculation of the maximum adsorption capacities of the adsorbents to optimize their use. The linearized form is as shown in Eq. (3).

(3) $$\frac{1}{q_{e}}=\frac{1}{q_{m}}+\frac{1}{K_{L}q_{m}C_{e}}$$

Where; q_e_ is the amount of ions adsorbed at equilibrium (mg/g), C_e_ is the concentration of the ions in the solution at equilibrium (mg/L), q_m_ is the maximum monolayer adsorption capacity of the adsorbent (mg/g), K_L_ is Langmuir constant related to the adsorption capacity (mg/g) that is calculated from the slope of the graph. A plot of 1/q_e_ against 1/C_e_ gives a straight line from which q_m_ and KL are obtained from the Y-intercept and slope, respectively. Also, the essential characteristics of the Langmuir isotherm can be expressed by a dimensionless constant called the separation factor RL calculated as shown in Eq. (4).

(4) $$R_{L}=\frac{1}{1+K_{L}C_{o}}$$

R_L_ values indicate the adsorption process is favorable when 0 < R_L_ <1 and unfavorable when R_L_>1.

#### 2.4.2 Freundlich isotherm model

This model assumes multilayer adsorption on heterogeneous adsorption sites (Ayawei et al., 2017). The linearized form of the model is as shown in Eq. (5).

(5) $$Logq_{e}=LogK_{f}+\frac{1}{n}LogC_{e}$$

Where; q_e_ is the amount of ions adsorbed at equilibrium (mg/g), C_e_ is the concentration of the ions in the solution at equilibrium (mg/L). A plot of Log qe against Log Ce gives a straight line from which the Value of Freundlich constant K_f_ and 1/n can be calculated from the Y-intercept and slope, respectively.

### 2.5 Desorption studies

The optimal 1.5g of rice husk biochar, coconut husk biochar and coffee husk biochar solution were used for adsorption, with initial nitrates concentrations of 204, 290 and 260 mg/L, respectively. The residues from equilibrium adsorption investigations were used to desorb nitrates. Similarly, the respective biochars added into a solution with initial nitrites concentrations of 6.09, 5.81 and 5.54 mg/L, respectively were desorbed. These desorption experiments were performed using deionized water. The centrifuge tubes containing 50 mL desorption solution were equilibrated for 150 min. At varying time intervals (30, 60, 90, 120, and 150 min), the supernatant was withdrawn with a syringe and filtered similarly as already described for batch adsorption studies to determine the concentration of the NO3-N and NO2-N. Before desorption experiments, the residues were oven-dried at 105 ᵒC for 12 h Eq. (6) used by Ajmal et al. (2020) in the study desorption studies was adopted for calculating the desorption efficiency of the materials in the study.

(6) $$Desorption\;Efficiency=\frac{C\times V}{q\times m}\times 100\%$$

Where; C (mg/L) is the amount of ions desorbed into the desorption solution, V (L) is the desorption solution volume, q (mg/g) is the equilibrium adsorbed amount of the ions before desorption, m (g) is the mass of the adsorbent used in desorption experiment.

## 3 Results and discussion

### 3.1 Materials characterization

The physical and chemical properties of the slaughterhouse wastewater used in this study are presented in Table 1.

**Table 1:** Physicochemical properties of slaughterhouse wastewater.

Parameter	Symbol	Unit	Value in this study	WHO-Limit
**Physicochemical characteristics**				
p H	-	-	7.35 ± 0.15	6.5–8.5
Electrical conductivity	EC	μS cm-1	1.39 ± 0.002	400 to 600
Chemical oxygen demand	COD	mg L^−1^	12800 ± 64	50
Biological oxygen demand	BOD	mg L^−1^	212.05 ± 15.16	30
Total solids	TS	mg L^−1^	1.371 ± 0.16	
**Macronutrients**				
Ammonia- nitrogen	NH_4_^+^-N	mg L^−1^	532.3 ± 0.1	100
Nitrate nitrogen	NO_3_-N	mg L^−1^	130.74 ± 0.1	10
Nitrite nitrogen	NO_2_-N	mg L^−1^	13.7 ± 0.1	10
Phosphate	PO_4_-P	mg L^−1^	5.93 ± 0.00	10
**Plant essential metals**				
Calcium	Ca	mg L^−1^	4.926	100
Copper	Cu	mg L^−1^	-	1
Zinc	Zn	mg L^−1^	0.178	0.5
Iron	Fe	mg L^−1^	0.902	10
**Plant non-essential metals**				
Lead	Pb	mg L^−1^	-	0.1
Nickel	Ni	mg L^−1^	-	0.05
Cadmium	Cd	mg L^−1^	0.009	0.005
Chromium	Cr	mg L^−1^	-	1

The quality of wastewater generated at the slaughterhouse highlighted major nutrients/micronutrients that are unfit for safe discharge into the environment, as the concentration level exceed the permissible discharge level prescribed by WHO (Kinuthia et al., 2020). Considering the low cost and efficiency of the technologies used, the adsorption of the nutrients investigated in this study contributed to the mitigation of their possible impacts if discharged to the environment untreated. From the elemental analysis result (Table 2), it can be observed that carbon contained the highest amount compared to other elements (H, N, and O). And which might indicate the dominance of carbon-containing functional groups.

**Table 2:** Physicochemical characteristics of the tested adsorbents.

Parameter	Rice Husk Char	Coconut Husk Char	Coffee Husk Char
**Physical characteristics**			
Pore diameter (nm)	1.97 ± 0.02	6.84 ± 0.015	1.63 ± 0.02
EC (μs cm)	170.5 ± 0.20	135.47 ± 0.153	150.40 ± 0.20
pH	9.37 ± 0.15	10.7 ± 0.20	8.94 ± 0.015
Bulk density (g/cm3)	0.16 ± 0.002	0.83 ± 0.002	0.296 ± 0.002
**Chemical characteristics**			
Ash (%)	53.47	3.33	4.54
Moisture (%)	9.04	6.57	4.42
Volatile matter (%)	27.33	13.22	29.92
Fixed carbon (%)	10.17	76.88	61.12
Carbon (%)	43.43	38.46	43.03
Hydrogen (%)	24.3	29.1	27.6
Nitrogen (%)	1.96	2.31	2.22
Oxygen (%)	22.63	30.62	24.36
**Reported composition of wastewater in different studies**			

	**Nitrate adsorption capacity (mg/g)**	**References**	**This study**
pH	7.4	(Istifanus and Bwala, 2017)	7.32
EC	1.5	(Apatie, 2016)	1.38
COD	12100	(Ewida, 2020)	12800
BOD	210	(Kariuki, 2014)	212.05
NH_4_^+^N	650		532.2
NO_3_^-^ N	115.5	(Aziz et al., 2018)	130.73
NO_2_^-^ N	14.7	(Huang et al., 2015)	13.6
PO_4_-P	7.0	(Anda et al., 2018)	5.93

Biochars can be graded into three classes based on their carbon content. According to Mohan et al. (2014), class 1 contains ≥60% carbon, class 2 carbon content lies between 30 and 60%, and class 3 contains between 10 and 30% carbon in the biochar. All the biochars used in this study have a carbon content in the range between 38 - 44% and therefore can be classified to be in class 2.

Table 2 shows how the contents of the major elements of the three biochars (rice, coconut, and coffee) varied depending on the feedstock and pyrolysis condition. From the results, it can be observed that the carbon percentages were higher than the percentages of hydrogen, nitrogen, and oxygen, respectively. The carbon constituent in the CoHB yielded the optimal treatment result during adsorption. An indication that their variations in adsorption capacities were based on their physical properties rather than chemical properties (Ambaye et al., 2020; Samer, 2015). Secondly, the hydrogen (H) constituent of the biochars showed a higher value for the CoHB (29.1%), followed by CcHB (27.6%) and RiHB (24.3%). And that the results obtained are in conformity with studies conducted by Kiggundu & Sittamukyoto (2019); Menya et al. (2018); Milla et al. (2013) and Bakshi et al. (2020) using agricultural feedstock for the treatment of nutrients in wastewater. The finding showed that the pyrolysis process has a systematic impact on the elemental compositions of each biochar produced from agricultural biomass. Thirdly, the oxygen (O) content in the analysis shows that CoHB (30.62 %) was greater than CoHB (24.36 %) and RiHB (22.63 %). Furthermore, light organic components, which are generally molecules that disintegrate into compounds containing high concentrations of hydrogen, are more destroyed during the pyrolysis process (light hydrocarbons and simple structure polymers) (Jahirul et al., 2012). This phenomenon contributes significantly to the materials' elemental makeup being reduced (Mierzwa-Hersztek et al., 2019). Lastly, the differences in values for the nitrogen (N) content, follow a conformed pattern with CoHB having the highest value of 2.31 %, CcHB (2.22 %), and RiHB (1.96 %). These results were in conformities with similar studies using agricultural biochars conducted by Zhao et al. (2017); Domingues et al. (2017) and Gai et al. (2014). From the results, it can be noted that the pyrolysis process has a significant impact on the characteristics of RiHB, CoHB, and CcHB. And that the drop in hydrogen and oxygen concentrations were mostly caused by the dissolution of oxygenated bonds and the release of H and O-containing low molecular weight by-products. Furthermore, it can be suggested that during pyrolysis, the volatile components were gradually eliminated due to a higher degree of polymerization.

**Figure 1:**
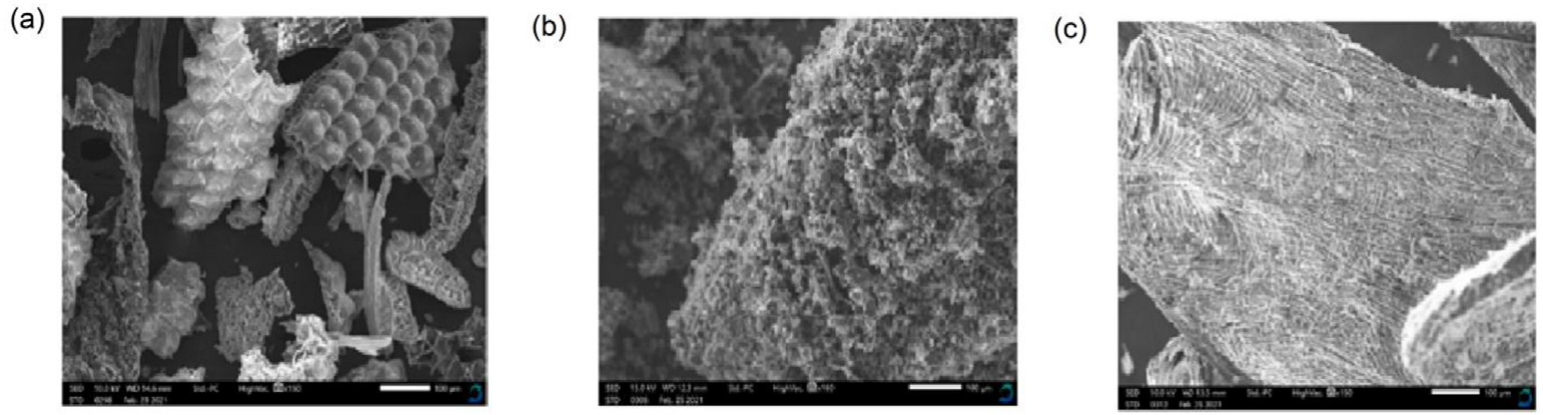
SEM images of rice husk char (a) coconut husk char (b), and coffee husk char (c).

The morphological properties of the adsorbents are presented in Figure 1. The surface adsorbent texture was visualized using SEM. The outer rice husk surface has a well-organized morphological structure that is corrugated in some places. With fewer pores, this could be due to the presence of inorganic elements as expressed by the high content of ash whose main constituents are a substantial amount of silica (SiO_2)_, Alumina (Al_2_O_3_) and ferric oxide (Fe_2_O_3_), as well as to alkalis of Na_2_O and K_2_O (Chindaprasirt et al., 2007). The appearance of the rice husk surface is also influenced by the cellulose-lignin matrix. The black patches are pore apertures and cavities, which may aid in solution flow and increase adsorption kinetics (Babiker et al., 2020; Jie et al., 2018). Coconut husk char exhibited a crystalline structure with multiple voids and micro-pores (Das et al., 2015). Coffee husk char although appeared crystalline, it had very few pores that were randomly distributed. Based on the morphology of the adsorbents, it can be deduced that coconut char was likely to have a large surface area for adsorption of ions since it was more porous (Chin et al., 2018; Song et al., 2014). Song et al. (2014) presented a similar result in their work, where coconut shell formed a richer pore structure that allowed for better adsorption of lead from aqueous solutions. However, the crystalline structure of the coffee husk and coconut husk chars could be an indication of having more cellulose content than the rice husk char (Grząbka-Zasadzińska et al., 2021).

**Figure 2:**
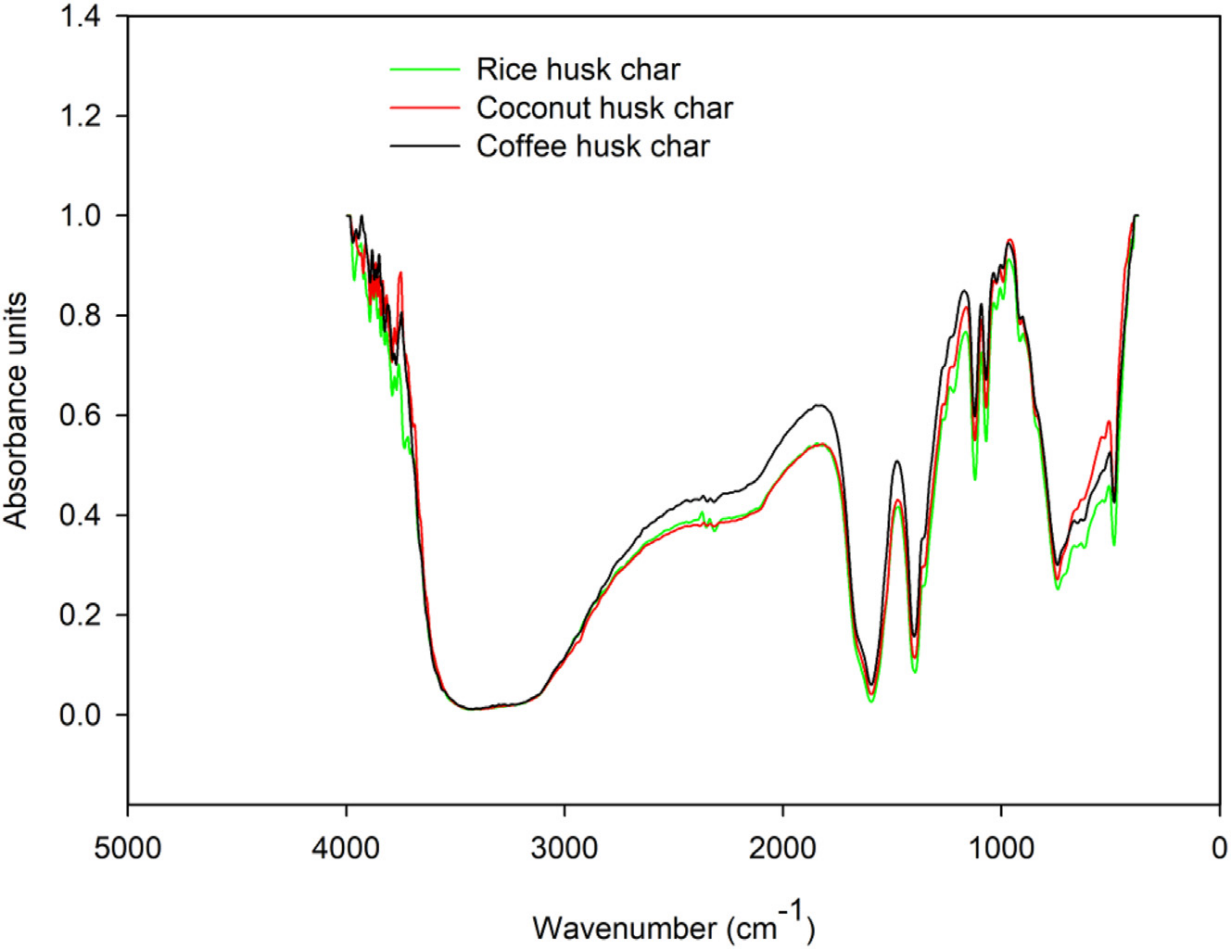
FTIR spectra of rice husk char, coconut husk char, and coffee husk char.

The FTIR spectra of rice husk char, coconut husk char and coffee husk char were less similar as depicted in Figure 2, an indication that their variations in adsorption capacities were based on their physical properties rather than chemical properties. In general, physical and chemical mechanisms control the sorption of nutrients by biochar. Chemical sorption is dependent on the type of biochar, the number and type of functional groups, and the chemical composition of the biochar, while physical sorption is related to the structure and surfaces of the biochar. A similar finding was reported by Nobaharan et al. (2021). The authors reported that the biochar physical properties were affected by the P sorption from wastewater, structural properties derived from different biochar original feedstocks. The peaks around 3600 cm^−1^ are attributed to the –OH stretching as a result of dehydration of the hemicellulose and cellulose (Fu et al., 2016). The broad peak between 2950-2800 cm^−1^ could be attributed to aliphatic methyl and methylene groups (Lun et al., 2017). The sharp peak around 1600 cm^−1^ is attributed to the stretching of the aromatic rings corresponding to the presence of alkene groups C=C (Liu et al., 2015). The dominantly sharp peaks around 1100 cm^−1^ may be attributed to the stretching of silicon containing functional groups (Si–O–Si) (Fu et al., 2016). Further investigation of the elemental analysis results reported in Table 4 reveals that carbon was present in higher levels than other elements (H, N, and O), possibly indicating the dominance of carbon-containing functional groups.

Table 4 shows the moisture contents of the rice (RiHB), coconut (CoHB), and coffee (CcHB) husks biochar. Studies conducted by Mikos et al. (2019); Palniandy et al. (2019) revealed that some biochars have a high hygroscopic tendency, allowing them to reabsorb water in their pores. And from the results obtained, the RiHB retained more moisture in its pores. And this led to its poor absorption performance in the removal of nutrients from slaughterhouse wastewater. Results obtained by Aller et al. (2017) indicate non-homogeneity due to the biochar samples generated from various types of biomass. Therefore, it can be concluded that the moisture content of the biochar from rice, coconut, and coffee husks play an important role in the removal of nutrient from wastewater. And that the higher the moisture content, the poorer its performance in the removal of nutrients, and the reverse. The application of biochar derived from rice husk and rubber wood biochar was studied by Palniandy et al. (2019). The finding suggested that the high moisture content level of rice husk biochar could be owing to biochar's hygroscopic nature after pyrolysis, which allows it to absorb moisture from the environment. The parameter of the volatile matter content of biochars is important in evaluating its combustion and adsorption effects (Sadiku et al., 2016). The volatile matter contains hydrocarbon compounds such as combustible or incombustible gas, or a mixture of both, that are emitted while biomass is burning, and this gas has a significant impact on biomass combustion behaviour (Suryaningsih et al., 2018). Also, studies conducted by Fernandes et al. (2020); Mierzwa-Hersztek et al. (2019); Suman & Gautam (2017); and Tomczyk et al. (2020) indicates that the thermal conversion of organic materials by pyrolysis varies and has a significant reduction of volatile matter content in biochar. As shown in Table 2. It may be concluded that the product of fixed carbon in biochar is strongly related to the volatile matter content, and the volatile matter concentration influences the fixed carbon content. That is, the higher the combustibility the higher will be the volatile matter content and the reverse. And also, the higher the combustibility the lower will be the fixed carbon content, and the reverse. Lastly, these results show that CcHB and RiHB were more combustible than CoHB. As indicated by Falemara et al. (2019), biomass with high mineral matter produces high ash content than those with low mineral matter during combustion. The high level of silica in some agricultural biomass like rice husk also encourages high ash content (Tomczyk et al., 2020). Additionally, study done by Lohri et al. (2015) shows that rice husks produce a significant proportion of ash in their natural state. Neina et al. (2020) also revealed that the high concentration of ash content is associated with an increase in EC values. In other studies, high ash content was found to harm the results of using biochar for nitrogen and phosphorus removal, as well as the sorption of ammonium and phosphate from aqueous solution by biochar generated from phytoremediation plants (Dai et al., 2020; Zeng et al., 2013). Therefore, from the results obtained in Table 2, it can be concluded that RiHB has higher mineral content than CoHB and CcHB during combustion. Also, the high ash content obtained in the RiHB was a result of the high level of silica as mentioned by Tomczyk et al. (2020).

The amount of ash in the biomass harmed the RiHB adsorption capability, causing it to perform poorly in adsorbing nutrients from slaughterhouse wastewater. Lastly, the amount of ash content present in the biochars can influence the result of the EC (viz., the higher the ash content concentration, the higher will be the EC, and the lower the ash content concentration the lower the EC value).

The fixed carbon of RiHB, CoHB and CcHB is presented in Table 2. CcHB and CcHB showed higher values compare to the RiHB. Biochar fixed carbon is proportional to its ash content during the pyrolysis process (Domingues et al., 2017). The authors further stated that the higher the ash content, the lower will be the fixed carbon yield. The presence of cellulose and hemicellulose at higher pyrolysis temperatures may have contributed to reduced rice husk degradation. In a nutshell, biomass with varying fractions of lignocellulose components decomposes differently, resulting in variations in the amount of fixed carbon generated (Bruun, 2011). A similar account presented by Dunnigan et al. (2016); Somparn et al. (2020); and Tomczyk et al. (2020) characterized the effect of process conditions on properties of biochar from agricultural residues and physicochemical characteristics of biochar from the pyrolysis process and feedstock type effects respectively. During the pyrolysis process, biochar underwent substantial breakdown, which was influenced by the pyrolysis condition thereby yielding minimal fixed carbon. Therefore, it can be concluded that the fixed carbon of biochar is directly proportional to the ash content. That is, the higher the fixed carbon content, the lower will be the ash content. And the lower the fixed carbon content, the higher will be the ash content.

### 3.2 Effects of biochar dosage, pH, and contact time on the amount of NO_3_^-^N and NO_2_^-^N removed and added

**Figure 3:**
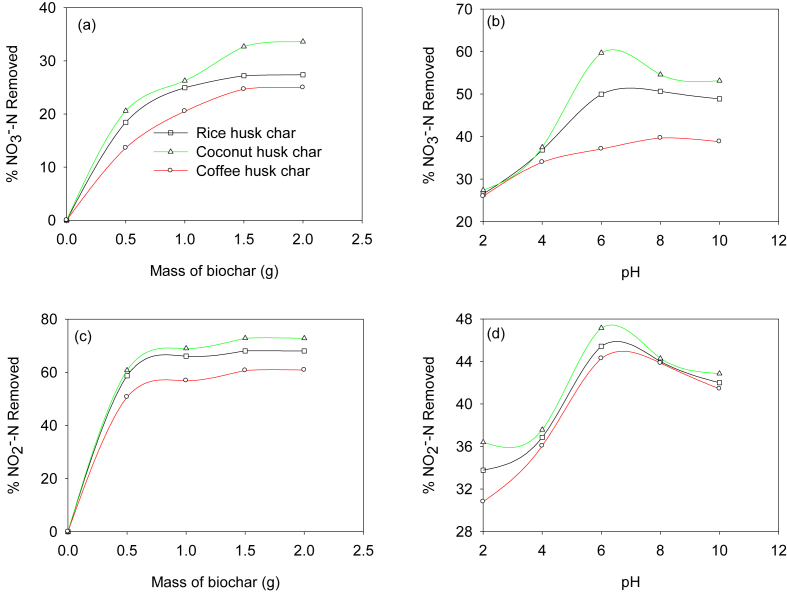
Effect of biochar mass and pH on NO_3_^-^N removal (a–b), and on NO_2_^-^N removal (c–d).

The effect of biochar dosage on the removal of NO3 -N and NO2 -N is presented in Figures 3a, c, respectively. It was observed that an increase in the mass of the biochar (viz., from 0.5g to 1.5g) led to an increase in the percentage of NO3-N and NO2 -N removal. Thus leading to a gradual decrease in removal of nutrients for biochar dosage >1.5g. As reported by Gorzin & Bahri Rasht Abadi (2018), this observation can be attributed to a gradual increase in available surface area for adsorption. However, the gradual reduction beyond 1.5g, can as well be attributed to the agglomeration of particles, thereby shielding the available adsorption sites. Similar findings on the effect of adsorbent dosage on the removal of anions from aqueous solutions were reported by Divband Hafshejani et al. (2016) and Liang et al. (2016).

**Figure 4:**
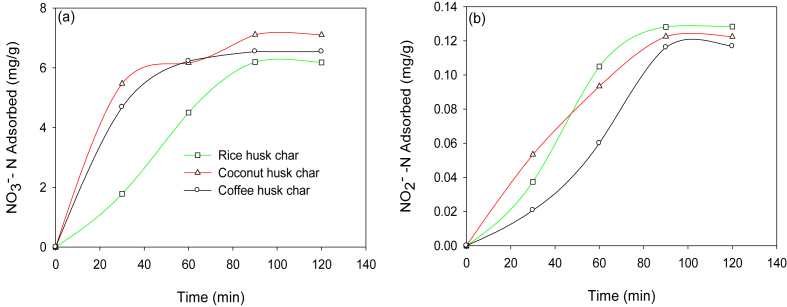
Effect of contact time on NO_3_^-^N (a), and NO_2_^-^N (b) adsorption at different initial concentrations.

Also, Figure 3b, d show the effect of initial solution pH on the adsorption of NO3 -N and NO2 –N, respectively. The pH varied from 2.0 to 10.0, while the adsorbent dosage was kept constant at 1.5g in 50.0 mL of solution. At pH of 2.0–4.0, the percentage removal was lower (Figure 3b, d). As the pH increases from 4.0 to 7.0, the percentage removal also increased. And at a pH of 7.0, the percentage removal gradually decreased. At lower solution pH, the surface of the biochars has positive charges due to the protonation reactions which consequently increases the electrostatic attraction between the biochar surface and negatively charged ions of nitrates and nitrites (Chintala et al., 2013). However, an increased pH value led to higher competition between the anions (viz., NO3 -N and NO2 -N) and hydroxide ions for the same adsorption sites (Cengeloglu et al., 2006). Lastly, the effect of the contact time on the adsorption guided this study in determining whether an equilibrium was attained within the duration of conducting the adsorption experiment or not. Contact time gives insights on the rate of uptake of the ions by the adsorbents with a rapid change of time in the adsorption process. The equilibrium adsorption of NO3-N and NO2- N was attained by all the biochars used in this study (Figure 4a, b, respectively). During the study, it was observed that the adsorption of the ions was initially rapid between 0 to 60 min, then gradually decreased between 60 to 90 min beyond unnoticeable adsorption. The initial rapid uptake can be attributed to the availability of vacant adsorption sites which gradually got occupied towards equilibrium, thereby slowing down the uptake of the ions. Other studies have attributed the variations in the rate of uptake of the ions with time to the existence of a high ionic gradient between the adsorption sites and the solutes in the solution at the initial stages of adsorption consequently resulting in high mass transfer (Kizito et al., 2015; Otieno et al., 2021).

### 3.3 Adsorption isotherm

#### 3.3.1 Equilibrium adsorption isotherm modelling of Nitrate Adsorption on Rice Husk Char, coconut husk char and coffee husk char

**Figure 5:**
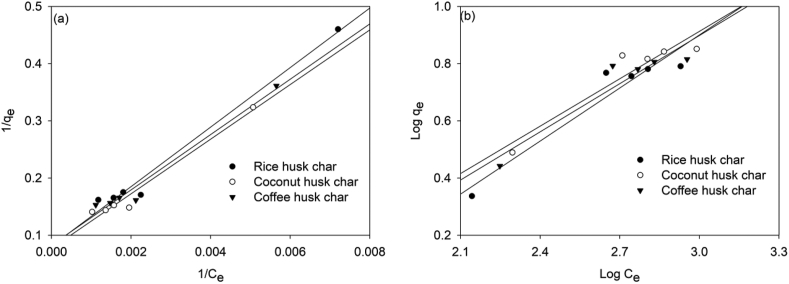
Langmuir plots (a), and Freundlich plots (b) for nitrate adsorption on rice husk char, coconut husk char and coffee husk char.

The linear regression plots for Langmuir and Freundlich models obtained for nitrates adsorption on the biochars are presented in Figures 5a, b, respectively. The adsorption of nitrates occurred in a monolayer on homogenous active sites. Based on the coefficient of correlation values (R2) obtained, it was observed that the Langmuir model best describe the adsorption of nitrates on the surface of rice husk char, coconut husk char and coffee husk char. Over time, the Langmuir model has been reported to be the best fit that describes nitrate adsorption onto various biochars derived from sugarcane bagasse, bamboo, and rice husks (Divband Hafshejani et al., 2016).

Also, the model parameters obtained from the Langmuir and Freundlich plots are presented in Table 3. Among the adsorbents used, coconut husk char had higher adsorption of nitrates. This high adsorption of nitrates property was attributed to its higher porosity as depicted in the SEM images. As a result, a larger surface area was available for the ions to attach themselves. However, the RL value for all the chars was in the range of 0.0–1.0 which is an indication that the conditions for nitrate adsorption were favorable. The differences in the initial solution concentrations, pH, solution temperature, and carbonization temperatures of the biochars may have contributed to the studies' adsorption capabilities.

**Table 3:** Adsorption isotherm model parameters for NO_3_^-^-N.

Isotherm	Parameter	Rice Hus Char	Coconut Husk Char	Coffee Husk Char
Langmuir	q _max_ (mg/g)	12.315	12.97	12.08
	K_L_	0.0015	0.0016	0.0017
	R_L_	0.382	0.342	0.348
	R^2^	0.983	0.971	0.973
Freundlich	K_f_	0.111	0.180	0.164
	1/n	0.618	0.552	0.562
	R^2^	0.917	0.894	0.897

#### 3.3.2 Equilibrium adsorption isotherm modelling of Nitrite Adsorption on Rice Husk Char, coconut husk char and coffee husk char

**Figure 6:**
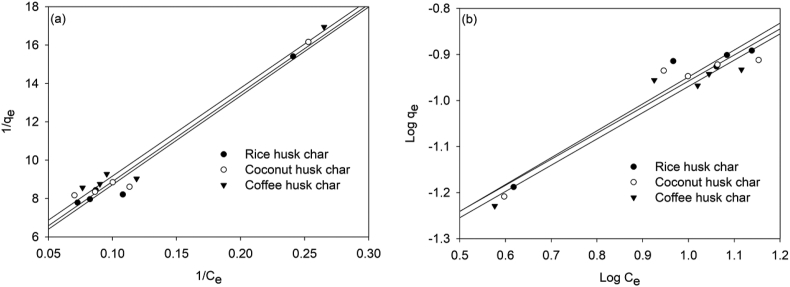
Langmuir plots (a), and Freundlich plots (b) for nitrite adsorption on rice husk char, coconut husk char, and coffee husk char.

Just like for nitrates, coconut husk char also revealed that it had a higher adsorption capacity of nitrites compared to rice husk char and coffee husk char, as presented in Figures 6a, b. A property attributed to its high porosity. However, the adsorption capacities of the chars for nitrites were substantially lower compared to nitrate adsorption. This could be attributed to the lower initial concentration of nitrites in the slaughterhouse wastewater. Other investigations have shown nitrate and nitrite adsorption capabilities on various biochars, which are similar to the circumstances in our experiment. For example, in a study conducted by Thao et al. (2021), rice husk biochar was found to have a nitrate adsorption capacity of 0.129 mg/g (Aghoghovwia et al., 2020), studied Pine wood biochar and found 15.2 mg/g, and (You et al., 2019) studied Coconut shell biochar and found 15.14 mg/g. However (Thao et al., 2021), investigated Rice husk biochar and found 0.2477 (mg/g) (Gierak and Łazarska, 2017), investigated Commercial carbon and found 0.2348 (mg/g), and (A Hanafi, 2016) investigated Activated carbon from rice straw and found 1.1 (mg/g). These results were predicated on the characteristics of chars in this paper, which have been extensively elucidated in section 3.1.

The isotherm model parameters calculated from the regression plots in Figure 6 are presented in Table 4.

**Table 4:** Adsorption isotherm model parameters for NO_2_^-^N adsorption.

Isotherm	Parameter	Rice Husk Char	Coconut Husk Char	Coffee Husk Char
Langmuir	q _max_ (mg/g)	0.233	0.244	0.218
	K_L_	0.088	0.093	0.099
	R_L_	0.345	0.344	0.339
	R^2^	0.976	0.973	0.974
Freundlich	K_f_	0.029	0.029	0.028
	1/n	0.584	0.566	0.570
	R^2^	0.939	0.918	0.929

### 3.4 Desorption studies

**Figure 7:**
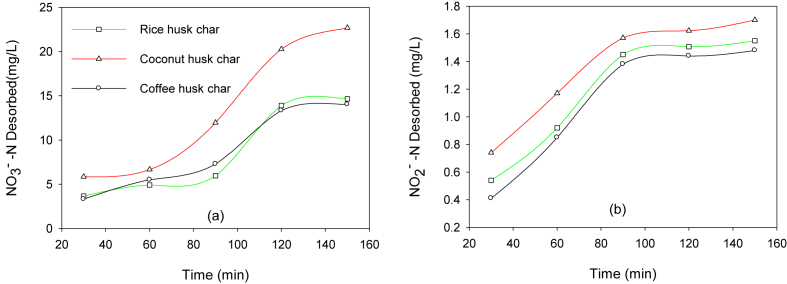
Desorption of NO_3_^-^N (a), and NO_2_^-^N (b) from rice husk char, coconut husk char and coffee husk char.

Figures 7a, b depict the rate of release of nitrates and nitrites in the solution, respectively. For nitrates, the desorption rate was observed to be slow between 30-90 minutes, then it increased rapidly between 90-120 minutes, beyond which no increase in solution concentration was observed. On the other hand, for nitrites, desorption was rapid from the onset at 30 minutes up to 90 minutes beyond which solution concentration was constant.

In order to gain understanding on the actual amount of nutrients released when desorption was constant, desorption efficiency was calculated using Eq. (6) and the results presented in Table 5.

**Table 5:** Desorption efficiencies of the tested adsorbents.

Desorption Efficiency (%)	Rice Husk Char	Coconut Husk Char	Coffee Husk Char
NO_3_^-^ N	22.4	24.39	16.79
NO_2_^-^N	80.73	91.39	83.62

The results portrayed that the desorption rate of nitrates from the adsorbents were 22.4, 24.39, and 16.79 %, for rice husk char, coconut husk char and coffee husk char, respectively. Similarly, Nitrites desorption rates from the adsorbents were 80.73, 91.39, and 83.62 %, for rice husk char, coconut husk char and coffee husk char, respectively. The desorption of nitrates and nitrites indicate that they were held by weak electrostatic forces on biochars and thus the enriched biochars could be used as slow-release fertilizers (Aghoghovwia et al., 2020). However, the rate of release of nitrites was significantly higher than for nitrates, an indication that they are easily leached. The reported information on desorption rates of NO3-N and NO2-N from biochar in this study are comparable with previous findings (Aghoghovwia et al., 2020; Ajmal et al., 2020). Overall, plants utilize nitrogen in the form of nitrates and ammonium (Dai et al., 2015) and therefore the slow-release of the nitrates observed in this study is beneficial to plants.

## 4 Conclusion

Biochar has received a lot of attention in recent years due to its unique structure and properties, coupled with its cost-effectiveness and environmentally friendly attributes. Various physical and chemical mechanisms control the sorption of nutrients by biochar in general. Chemical sorption is dependent on the type of biochar, the number and type of functional groups, and the chemical composition of the biochar, while physical sorption is related to the structure and surfaces of the biochar. This study aimed at the removal of nitrates and nitrites from slaughterhouse wastewater using biochar derived from rice husk, coconut husk and coffee husk and also determine their desorption capacities. However, coconut husk biochar exhibited a higher adsorption capacity of both nutrients a property attributed to its higher porosity as revealed by the SEM images. Because the Langmuir model provided the greatest fit, isotherm analyses demonstrated that the adsorption of both nutrients on all chars was monolayer adsorption on homogeneous active sites. It was also discovered that increasing adsorbent dosage, pH, or contact time increased nutrient adsorption up to a point where adsorption declined in the case of dosage and pH but stayed constant in the case of contact time. All of the biochars were able to gently release nitrates into the water solution, implying that they might be used as slow-release fertilizers. Overall, the findings show that biochars can take nutrients from slaughterhouse effluent and can thus be enriched to be utilized as organic fertilizers while also preventing the enrichment of receiving water bodies.

## Declarations

### Author contribution statement

Morris Konneh: Conceived and designed the experiments; Performed the experiments; Analyzed and interpreted the data; Contributed reagents, materials, analysis tools or data; Wrote the paper.

Simon M. Wandera, Sylvia I. Murunga & James M. Raude: Conceived and designed the experiments; Performed the experiments; Analyzed and interpreted the data; Contributed reagents, materials, analysis tools or data.

### Funding statement

This research did not receive any specific grant from funding agencies in the public, commercial, or not-for-profit sectors.

### Data availability statement

Data will be made available on request.

### Declaration of interests statement

The authors declare no conflict of interest.

### Additional information

No additional information is available for this paper.
